# Consensus guidelines for diagnosis and management of anemia in epidermolysis bullosa

**DOI:** 10.1186/s13023-022-02448-w

**Published:** 2023-02-23

**Authors:** Carmen Liy-Wong, Cristina Tarango, Elena Pope, Thomas Coates, Anna L. Bruckner, James A. Feinstein, Agnes Schwieger-Briel, Lynne D. Hubbard, Clapham Jane, Mauricio Torres-Pradilla, Matija Zmazek, Irene Lara-Corrales

**Affiliations:** 1 Division of Dermatology and Rheumatology, Children’s Hospital of Eastern Ontario, University of Ottawa, Ottawa, ON Canada; 2 Division of Hematology, Cancer and Blood Diseases Institute, Cincinnati Children’s Hospital Medical Center, University of Cincinnati College of Medicine, Cincinnati, OH USA; 3 Division of Dermatology, The Hospital for Sick Children, University of Toronto, Toronto, ON Canada; 4 Keck School of Medicine, Division of Hematology/Oncology, Department of Pediatrics, Children’s Hospital Los Angeles, University of Southern California, Los Angeles, CA USA; 5 Epidermolysis Bullosa Clinic, Children’s Hospital Colorado, University of Colorado School of Medicine, Aurora, CO USA; 6 Pediatric Skin Center, Department of Dermatology, University Children’s Hospital Zurich, Zurich, Switzerland; 7 Department of Nutrition and Dietetics, Rare Diseases Centre at St Thomas’ Hospital, Guy’s and St Thomas’ NHS Foundation Trust, London, UK; 8 Lead EB CNS, Rare Diseases Centre, St John’s Institute of Dermatology, St Thomas’ Hospital, Guy’s and St Thomas’ NHS Foundation Trust and DEBRA UK, London, UK; 9 Fundación Universitaria de Ciencias de la Salud and Hospital de San José, Bogotá, Colombia; 10 DEBRA, Zagreb, Croatia

**Keywords:** Epidermolysis bullosa, Anemia, Recessive dystrophic epidermolysis bullosa, Iron deficiency, Chronic anemia of inflammation

## Abstract

**Background:**

Anemia is a common complication of severe forms of epidermolysis bullosa (EB). To date, there are no guidelines outlining best clinical practices to manage anemia in the EB population. The objective of this manuscript is to present the first consensus guidelines for the diagnosis and management of anemia in EB.

**Results:**

Due to the lack of high-quality evidence, a consensus methodology was followed. An initial survey exploring patient preferences, concerns and symptoms related to anemia was sent to EB patients and their family members. A second survey was distributed to EB experts and focused on screening, diagnosis, monitoring and management of anemia in the different types of EB. Information from these surveys was collated and used by the panel to generate 26 consensus statements. Consensus statements were sent to healthcare providers that care for EB patients through EB-Clinet. Statements that received more than 70% approval (completely agree/agree) were adopted.

**Conclusions:**

The end result was a series of 6 recommendations which include 20 statements that will help guide management of anemia in EB patients. In patients with moderate to severe forms of EB, the minimum desirable level of Hb is 100 g/L. Treatment should be individualized. Dietary measures should be offered as part of management of anemia in all EB patients, oral iron supplementation should be used for mild anemia; while iron infusion is reserved for moderate to severe anemia, if Hb levels of > 80–100 g/L (8–10 g/dL) and symptomatic; and transfusion should be administered if Hb is < 80 g/L (8 g/dL) in adults and < 60 g/L (6 g/dL) in children.

**Supplementary Information:**

The online version contains supplementary material available at 10.1186/s13023-022-02448-w.

## Background

Epidermolysis bullosa (EB) is a rare genetic disorder characterized by skin fragility with blister formation occurring spontaneously or following minor trauma such as gentle pressure or friction. It can be broadly divided into four major subtypes: EB simplex (EBS), junctional (JEB), dystrophic (DEB), and Kindler EB (KEB, previously known as Kindler syndrome) [1]. EB can affect multiple body systems, particularly the skin, mucosae, and sometimes internal organs. Subtypes are determined by several factors including the level of skin cleavage, phenotype, mode of inheritance, and molecular origin. Complications of severe forms of EB, mainly JEB and RDEB, almost invariably lead to chronic malnutrition and chronic anemia of inflammation, jeopardizing patients’ immune status, leading to poor growth, bone loss, delayed wound healing, and having a great impact on quality of life [1].

Although anemia in EB patients is a well-known entity, the numbers of published studies in this area are small, and clear guidelines outlining its optimal management do not exist. Anemia commonly occurs in patients with severe types of EB, but it also affects to a lesser extent other subtypes [2–4]. Profound iron deficiency contributes to chronic fatigue, reduced energy levels, dyspnea, poor exercise tolerance, impaired wound healing, and anorexia that are compounded by the presence of anemia [4]. Many patients with severe forms of EB have hemoglobin levels of < 80 g/L despite therapy with iron supplements, and require blood transfusions to correct their anemia [4]. Prevalence of anemia varies in different EB subtypes. In 169 Australian pediatric and adult EB patients was 27.8% (47/169) at some point in their lifetime. Of those, anemia was present in 11.3% (9/80) of EBS, 37.5% (6/16) of JEB and 68.0% (17/25) of RDEB patients [5]. The prevalence of anemia did not differ significantly according to sex in adult groups, however the prevalence of anemia among adults were higher than those among pediatric patients [5]. Prevalence of anemia in Peruvian EB patients was reported to be 62.4% (58/95); of those, anemia was present in all RDEB patients, 62.5% (5/8) of JEB and 30% (12/39) of EBS [6]. Another report in London demonstrated anemia in 96% (52/54) of RDEB patients [7].

The etiology of anemia in EB is multifactorial, with chronic inflammation and iron loss being the primary factors. Due to extensive blistering, it is possible that JEB and RDEB patients lose more skin cells and have more direct blood loss than EBS patients, leading to increased iron loss [8]. In fact, iron is required for the replacement of the intestinal lining, so severe iron deficiency itself is associated with protein losing enteropathy and malabsorption of nutrients including iron, creating a vicious cycle perpetuating the anemia [9]. Severe chronic iron deficiency has been potentially implicated in loss of GI tract integrity resulting in ulcerations and esophageal strictures, the so-called Plummer-Vinson syndrome, but the mechanism remains unclear [10]. Involvement of the gastrointestinal tract produces ulceration, sloughing of the mucosa, inflammation, and bleeding, also contributing to iron loss [10]. Poor gastrointestinal mucosal integrity may decrease dietary iron absorption [4]. A recent study on oral iron challenge tests in RDEB patients demonstrated that (75%) 9/12 patients who received an oral iron dose did not show iron absorption [9]. Patients with RDEB often have scarring and strictures of the esophagus leading to poor dietary intake, especially of iron-rich foods such as meat, which may be difficult to swallow [4]. Finally, chronic wound healing and low-grade skin infections in EB patients produce a chronic inflammatory state leading to anemia of inflammation, in which there is inefficient utilization of iron in hematopoiesis. Inflammatory cytokines and an associated increase in hepcidin levels result in poor enteric iron absorption, inefficient utilization of iron and decreasing erythropoiesis [11]. Screening for iron deficiency anemia is problematic in EB patients. Values for ferritin, total iron‐binding capacity, transferrin receptor and free erythrocyte protoporphyrin correlate very poorly with hemoglobin (Hb), with only marginally better correlation with transferrin saturation and serum iron levels. There is also a weak inverse relationship between Hb levels, erythrocyte sedimentation rate (ESR) and serum albumin. These findings suggest that nearly all patients with severe EB have anemia of inflammation that can be accompanied by iron deficiency at some point [7]. Moreover, ferritin levels, which are markers of inflammation are often elevated in EB patients and therefore poor indicators of true iron storage [4]. One alternative is to use the ratio of soluble transferrin receptor (sTfR)/log ferritin (nl < 1) which can take into account the contribution of chronic inflammation [12].

Early management of iron deficiency and anemia is essential to reduce fatigue and other anemia related symptoms, to promote wound healing, enhance linear growth, and ultimately optimize quality of life. Current strategies involve improving nutritional status, oral or intravenous iron replacement, transfusing blood products, and administering erythropoietin [4]. Treatment of anemia with oral iron supplementation is broadly used in EB centers around the world but its individual effectiveness varies. Moreover, enteral iron may not be absorbed, or well- tolerated due to unpalatable taste, abdominal pain, constipation, and nausea [4, 9]. Intravenous iron and erythropoietin were beneficial in a small study of patients with RDEB who failed to respond to oral iron [13]. Blood transfusions should be considered for cases where Hb levels are consistently below 80 g/L and/or for symptomatic patients who do not respond to other measures [4]. Recently, a clinical algorithm for the diagnosis and treatment of anemia in RDEB patients was proposed: (i) start enteral iron if Hb ≤ 100 g/L after performing an enteral iron absorption test, as this may help to determine whether parental iron is indicated instead, (ii) consider IV iron if Hb is between 80 and 100 g/L and, (iii) consider blood transfusion if Hb is < 80 g/L [14]. However, this is an algorithm suggested by one institution, and every patient’s case should be individually decided.

## Objectives

The objective of this guideline development was to generate a list of recommendations that will allow practitioners to better manage anemia in EB patients.

## Methodology

### Systematic literature search and appraisal

Searches were performed with PubMed, Embase and Cochrane databases was using the terms ‘EB and anemia, ‘EB and iron deficiency’ and ‘EB and chronic anemia’, with no language restrictions and the search period ending in September 2020. In addition, ‘epidermolysis bullosa’ was used to search articles in GeneReviews. In total, 88 articles were identified. Of those, only 10 articles addressed anemia directly. No meta-analyses, systematic reviews, or case–control studies were available. The highest level of evidence was achieved by observational and cohort studies. Ten papers were appraised, each by two panel members. Due to the limited data available, a consensus expert opinion was preferred to develop the guidelines. Patients concerns and opinions were also taken into consideration for this consensus guideline. Using an online-based modified Delphi method for generating consensus [15], the list was translated into 2 surveys.

### Delphi process

#### Phase 1: Recruitment and establishment of guideline panel

In 2016, DEBRA International (DI) consulted with the international EB community and identified Clinical Practice Guideline (CPG) for Anemia in EB as 14th in the priority list to develop. A guideline panel was recruited consisting of 11 international experts in the field of EB, including a dermatologist, pediatric dermatologist, hematologist, pediatrician, EB dietician, clinical nurse specialist and patient representatives. All panel members completed written conflict of interest and code of conduct declarations. The consensus guideline development of clinical recommendations was led by two panel members (IL-C y CL-W).

#### Phase 2: Survey for EB patients and carers

A survey developed by the panel included 13 multiple-choice questions [Additional file 1], with the option for free-text elaboration in some questions. The questions were targeted at understanding self-recognition of anemia and treatment preferences in EB patients. Surveys were distributed via the EB CLINET newsletter, DEBRA International Patients’ forum, and to participants attending the EB 2017 Research Conference. A statement at the beginning of each survey informed participants that their responses were voluntary and anonymous, and completion would signify their informed consent. The only demographic data collected included whether the responders of the first survey were patients with EB or family members, and the type of health care provider, to help maintain confidentiality. Surveys were open for 6 months.

#### Phase 3: Primary panel meeting

The first face-to-face meetings, with nine panel members attending in person and two via teleconference, occurred to discuss the methodology and development of PICO questions [16] which defines a specific population (P), intervention (I), comparator (C), and outcome (O), according to the highest priority and results from the first survey were reviewed during the first panel meeting, at the EB 2017 Research Congress and 4th conference of EB Clinet held in Salzburg, Austria. Whenever input from the entire group was required, it was solicited via e-mail or the DEBRA international (DI) clinical practice guideline (CPG) coordinator (KM-C) facilitated communications.

#### Phase 4: Healthcare provider survey

A second survey was developed and reviewed by the panel members. It included 26 multiple-choice questions [Additional file 2] with the option for free-text elaboration and was aimed at understanding the clinical diagnosis, classification, and treatment of anemia in EB. This survey was targeted to healthcare providers looking after patients with EB. The results of the Healthcare Provider survey can be provided to readers by request.

#### Phase 5: Recommendation panel meeting

The final face to face meeting, with 8 panel members attending in-person and two via teleconference at the 2018 DEBRA International Congress in Zermatt, Switzerland, was convened to review the evidence, agreed consensus statements, structure and wording of the CPG. Panel members not present provided their input via minutes circulated in e-mails. During this meeting panel members reviewed survey results and drafted the guideline statements.

#### Phase 6: Consensus survey

The consensus survey included 26 statements drafted by the panel members. The healthcare providers (HCP) were asked to rate each recommendation on a 4-point Likert scale (strongly disagree, slightly disagree, slightly agree, and strongly agree) [17]. At least 70% agreement was required for each item to be adopted in the final list of recommendations, this decision was made by the expert panel members.

The surveys were circulated like previously via EB Clinet and DI as well as direct emails to (i) HCP caring for EB patients around the world, (ii) DEBRA countries in North and South America, Europe, and Australia in 2018 and (iii) participants attending the 2018 DEBRA International Congress.

#### Phase 7: Editorial

The final guideline draft was reviewed by the whole panel and all input was addressed. To increase the overall generalizability, an external panel representative of a cross-section of EB multidisciplinary team specialists (nine) and people living with EB (two) peer-reviewed the draft, and the Appraisal of Guidelines for Research & Evaluation (AGREE II) tool [18] was conducted by the DI CPG coordinator. The panel addressed all feedback in the final editing stage.

## Results

### Patient and family member survey

Out of 18 participants, 12 (66.7%) were EB patients and 6 (33.3%) were family members of EB patients. Seventeen of 18 specified their or their family member’s type of EB. Eleven of 17 (64.7%) had dystrophic EB, 5/17 (29.4%) had EB simplex and 1/17 (5.9%) had junctional EB. 12/ 18 participants (66.7%) worried about having anemia and 15/18 (83.3%) were able to recognize it when they became anemic by the following symptoms: fatigue and tiredness 13/15 (86.7%), low mood 7/15 (46.7%), slower wound healing 7/15 (46.7%) shortness of breath 6/15 (40%) and tachycardia in 5/15 (33%). Eleven out of 18 (61.1%) patients previously received treatment for anemia. The interventions used were oral iron in 8/11 (72.7%), dietary modifications 7/11 (53.6%), iron infusion 4/11 (36.4%), red blood cell transfusions 3/11 (27.3) Five out of 9 (55.6%) patients who responded to this section preferred oral iron treatment while 4/9 (44.5%) preferred iron infusions. Seven out of 14 of patients who responded to this section (50%) reported side effects from anemia treatment. The most common was constipation in 4/7 (57.1), followed by stomach pain in 3/7 (42.8%), dyspepsia in 2/7 (28.6%), allergic reaction in 2/7 (28.6%), nausea and vomiting in 1/7 (14.2%), and chest pain in 1/7 (14.2%).

### Consensus survey

Eighty-nine responders from 18 different countries responded to the survey. All continents were represented except Africa and Antarctica. Thirty-two surveys were excluded (31 were incomplete and 1 was completed by a parent), with 57 surveys included in the final analysis. The responders included 28 dermatologists, 12 pediatricians, 5 hematologists, 4 nurses, 3 pediatric dermatologists, 1 internist, 1 dietician, 1 physical therapist, 1 research fellow and 1 physician working with EB. Their experience working with EB patients varied, with 22/57 (38.6%) having 1–10 years of experience, 25/57 (43.9%) with 10–20 years and 10/57 (17.5%) with more than 20 years of experience. Thirty five of 57 (61.4%) HCPs reported seeing 1–10 EB patients per month, 13/57 (22.8%) see 10–20 per month, and 9/57 (15.8%) see more than 20 per month. This survey initially included 26 statements created by the panel members, of those 20 statements met criteria for inclusion with more than 70% of responders who strongly agreed or agreed with the statement, 6 statements did not meet criteria and were removed. A summary of recommendations is shown in Table 1.

**Table 1:** Summary of recommendations

Desirable consequences probably outweigh undesirable consequences in most settings, for this reason we suggest offering these options:	Consensus agreement in percentage (%)	References
**We recommend screening for Anemia in EB**		
1. For clinical suspected severe or generalized forms of EB, anemia should be ruled out at the age of diagnosis	95	5, 6, 7, 17, 20, 21
2. For moderate types of EB, anemia screening should start at 1 year of age	84	
3. For EB simplex anemia, screening should be done only if symptomatic	81	
**We recommend for the diagnosis of anemia in EB patients**		
1. That diagnosis and severity of anemia should be based on the WHO recommendations	91	22
2. An evaluation/assessment of anemia in EB requires a careful history and physical exam looking at potential causes including:	97	
(i) Diet, (poor oral intake, lack of protein in the diet)		
(ii) Gastrointestinal (GI) symptoms (mouth blistering and erosions, difficulty swallowing due to esophageal stenosis, stomach pain, diarrhea, constipation)		
(iii) History of pica or pagophagia (i.e., compulsive consumption of ice)		
(iv) Signs of blood loss (e.g., wound bleeding, epistaxis, menorrhagia, melena, hematuria, hematemesis)		
(v) Surgical history (e.g., esophageal dilatation, hand surgery)		
**We recommend treatment of anemia in EB**		
1. In patients with moderate to severe forms of EB, the minimum desirable level of Hb is 100 g/L (10 mg/L)	80	15, 23
2. Iron infusion should be administered in moderate to severe forms of EB, if Hb levels of 80–100 g/L (8-10 g/dL) and symptomatic	86	
3. For patients with moderate to severe forms of EB who failed iron infusion, transfusion should be considered	90	
4. For patients with severe forms of EB, transfusion should be administered if Hb < 80 g/L (8 g/dL) in adults and < 60 g/L (6 g/dL) in children**	78	
**We recommend monitoring biochemistry for anemia in EB, using**		
1. The gold standard for diagnosis of anemia is Hb level	90	20, 21, 23, 26*, 27*
2. Ferritin level can support diagnosis of iron deficiency. (Ferritin can be unreliable since it is also an acute phase reactant and will be high in states of inflammation, masking iron deficiency)	83	
3. If total iron-binding capacity (TIBC) is available, it can be useful to assess iron deficiency (if high it indicates iron deficiency)	71	
4. Patients with severe forms of EB require regular screening at fixed intervals every 6 months or at any time when symptomatic	93	
5. Laboratory testing that may be pertinent in the initial evaluation of anemia in EB patient should, in addition to the above, include	93	
(i) Complete blood count (CBC): hematocrit, mean corpuscular volume (MCV), mean corpuscular hemoglobin (MCH) and mean corpuscular hemoglobin concentration (MCHC)		
(ii) Reticulocyte count		
(iii) Iron profile (Includes serum iron, ferritin, total iron-binding content (TIBC) and soluble transferrin receptor if locally available)		
(iv) CRP		
**We recommend other treatment options to manage anemia in EB patients, include**		
1. Dietary measures should be offered as part of the management of anemia in all EB patients	95	2,4,7 8, 13,14, 21, 22*, 25*, 31, 30, 32, 34*, 36*, 39*, 40, 41*, 43*
2. Optimization of iron-rich food according to geographic location should be offered as part of the management of anemia in all EB patients	93	
3. Oral iron preparation that is readily available in each geographic area and that is tolerated by the patient should be the iron of choice	93	
4. Oral iron supplements should be administered every other day to maximize the absorption and minimize the side effects for all patient with mild to moderate anemia	70	
5. Oral iron should be administered for at least 4 weeks before assessing clinical benefit	86	
**We recommend the outcome(s) of treating anemia in EB patients should include**		
1. Improvement of symptoms (more energy, less fatigue, adequate wound healing)	98	
2. Improvement of laboratory parameters	89	

*References not in EB population

**The main goal of transfusion is to correct or avoid imminent inadequate oxygen carrying capacity caused by inadequate red blood cell mass. Although most anemia guidelines suggest transfusion when Hb level is < 80 g/L, the consensus panel acknowledges that children with EB can tolerate lower levels of Hb and transfusion in this pediatric population should be administered when symptomatic or when Hb ≤ 60 g/L

### Recommendation 1

#### At what age should we start looking for anemia in EB patients?

1. For clinical suspected severe or generalized forms of EB, anemia should be ruled out at the age of diagnosis.
2. For moderate types of EB, anemia screening should start at 1 year of age.
3. For EB simplex, anemia, screening should be done only if symptomatic.

The initial step in diagnosis and clinical management of anemia in EB patients must be the acquisition of laboratory and treatment data at specified intervals. Severity of anemia varies amongst different types of EB, being more common in RDEB (96–100%) and JEB (62.5–100%) but it can also affect less commonly patients with EB simplex (11–30%) [5–7]. The laboratory tests and the frequency of these tests vary depending upon the type of EB and the presence of complications [19]. During the first 1–2 years of life, it would be prudent to have any child with generalized EB seen at least every 6 months by a pediatrician or pediatric dermatologist [19]. A recent study that included 200 children, 157 with recessive dystrophic EB (RDEB) and 43 with junctional EB (JEB)‐generalized intermediate, followed at the main referral centre in Germany demonstrated anaemia was present from the second year of life onwards in RDEB and JEB. Low levels of Hb, iron, vitamin D, zinc and albumin, high levels of C‐reactive protein, and absence of collagen VII correlated significantly with low weight in RDEB. No correlation was observed in JEB [20].

### Recommendation 2

#### How should we diagnose anemia in EB patients?

1. Diagnosis and severity of anemia will be based on the WHO recommendations.

According to the World Health Organization (WHO), anemia is defined as Hb levels < 12.0 g/dL in women and < 13.0 g/dL in men (see Table 2). However, normal Hb distribution varies not only with sex but also with age, ethnicity, and physiological status. New lower limits of normal Hb values have been proposed, according to ethnicity, gender, and age. Anemia is often multifactorial and is not an independent phenomenon. For the classification and diagnosis, the hematologic parameters, the underlying pathological mechanism, and patient history should be considered. In general, anemia can be further classified as mild anemia with Hb 100–120 g/L, moderate anemia Hb 80–100 g/L, severe anemia Hb < 80 g/L [21].

2. To evaluate anemia in EB patients, a careful history and physical exam looking at potential causes is recommended.

**Table 2:** Hemoglobin levels to diagnose anemia at sea level (g/L)* according to WHO [21]

Population	Anemia			
	Non-anemia	Mild^a^	Moderate	Severe
Children 6–59 months of age	110 or higher	100–109	70–99	Lower than 70
Children 5–11 years of age	115 or higher	110–114	80–109	Lower than 80
Children 12–14 years of age	120 or higher	110–119	80–109	Lower than 80
Non-pregnant women (15 years of age and above)	120 or higher	110–119	80–109	lower than 80
Pregnant women	110 or higher	100–109	70–99	Lower than 70
Men (15 years of age and above)	130 or higher	110–129	80–109	Lower than 80

Adapted from reference [21], these cut-off levels were established in a non-EB population

*Hemoglobin in grams per litre

^a^“Mild” is a misnomer: iron deficiency is already advanced by the time anemia is detected. The deficiency even when no anemia is clinically apparent

The history should focus on potential aetiologies and may include questions about diet, (poor oral intake, lack of protein in the diet), gastrointestinal (GI) symptoms (difficulty swallowing due to esophageal strictures, abdominal pain, diarrhea, constipation), signs of blood loss (e.g., wound bleeding, epistaxis, menorrhagia, melena, hematuria, hematemesis), and surgical history (e.g., esophageal dilatation, hand surgery). According to the anemia patients’ survey, most patients (83%) were able to recognize when they became anemic, with fatigue and tiredness being the most common symptoms reported followed by low mood, slower wound healing, shortness of breath, and less commonly tachycardia. These symptoms should be asked about at each patient’s visit. In children we need to rely on parental report or blood work results since it is difficult to capture from reported symptoms alone.

### Recommendation 3

#### What should be the threshold for treatment of anemia?

1. In patients with moderate to severe forms of EB, the minimum desirable level of Hb is 100 g/L (10 mg/dL).
2. Iron infusion should be administered in moderate to severe forms of EB if Hb levels are 80–100 g/L (8–10 g/dL) and symptomatic.
3. For patients with moderate to severe forms of EB who failed iron infusion, transfusion should be considered.
4. For patients with severe forms of EB, transfusion should be administered if Hb is < 80 g/L (8 g/dL) in adults and < 60 g/L (6 g/dL) in children.

**Fig. 1:**
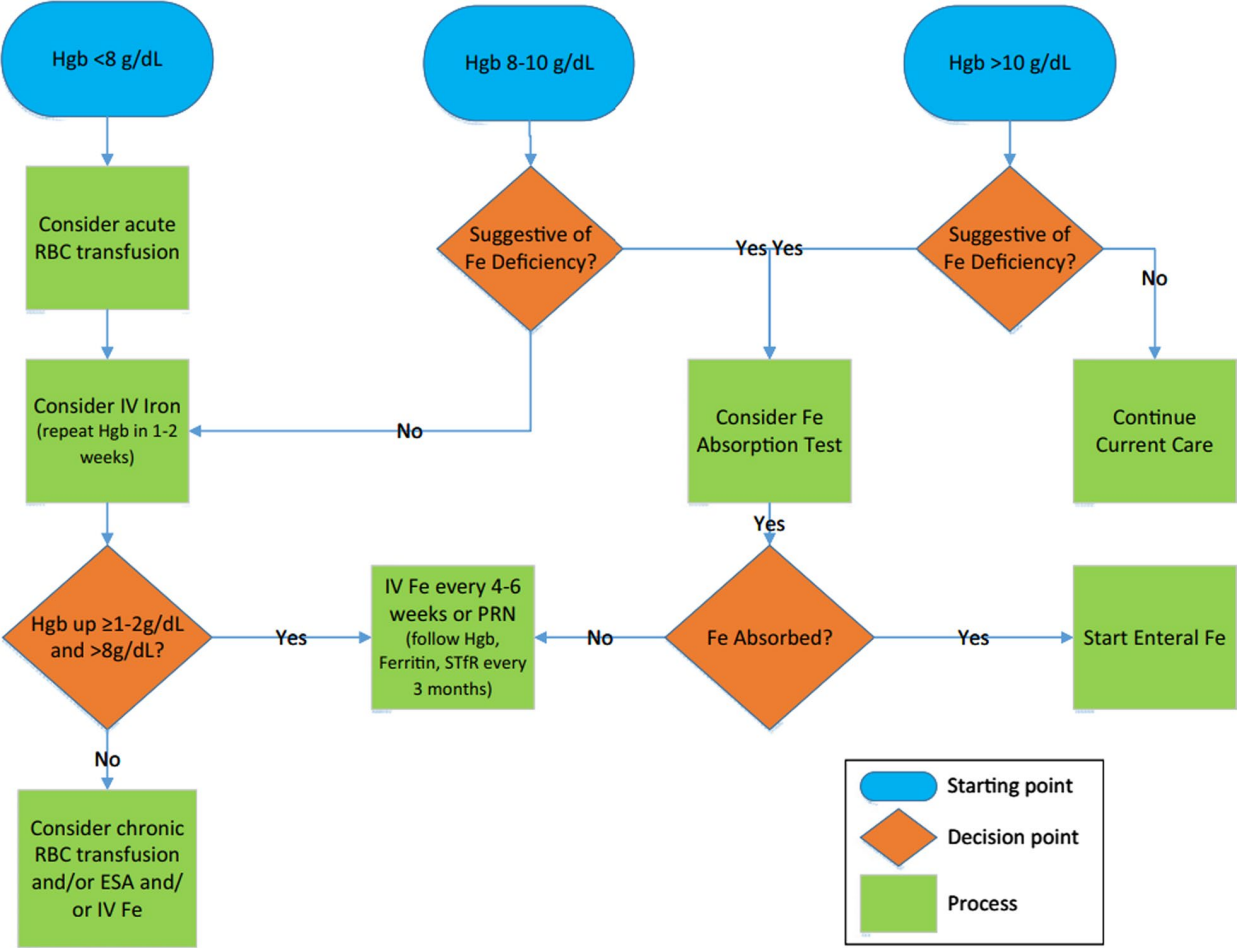
Epidermolysis bullosa anemia flowchart. Hgb, hemoglobin; Fe, iron; RBC, red blood cell; IV, intravenous; ESA, erythropoietin stimulating agent; STIR, soluble transferrin receptor, PRN, as needed [14]

The threshold for initiating treatment and the goals of treatment are subject to variation according to discipline and medical condition [22]. To guide the providers of patients with RDEB and help standardized the management of anemia, a group of experts in EB from Cincinnati Children’s Hospital developed a clinical algorithm to manage anemia in epidermolysis bullosa [14]. (Fig. 1).

### Recommendation 4

#### What are the best parameters for monitoring anemia in EB patients and how often do we need to obtain them?

1. The gold standard for diagnosis of anemia is hemoglobin level.
2. Ferritin level, if low, can support diagnosis of iron deficiency.
3. If total iron-binding capacity (TIBC) is available and elevated, it can indicate iron deficiency.
4. Patients with severe forms of EB require regular screening at intervals of every 6 months or at any time when symptomatic.

A recent study of 200 children, 157 with RDEB and 43 with JEB‐generalized intermediate, followed at the main referral centre in Germany demonstrated that anaemia was present in 91% of children with RDEB and 75% with JEB from the second year of life onwards [20]. With age, haemoglobin levels decreased further in RDEB, but improved towards adulthood in JEB. Serum iron levels, ferritin and transferrin saturation were below normal in half of children with RDEB aged 2–10 years and in > 80% of those aged > 10 years [20]. The mean laboratory parameters for anemia were reported as show in Table 3.

**Table 3:** Laboratory parameters in 200 EB patients [20]

Mean laboratory parameters	RDEB N = 157	JEB generalized intermediate N = 43
HB (g/L)	9.7 ± 2.23	11.1 ± 2.6
Reticulocytes (%)	17.8 ± 16.3	14.4 ± 12.9
Ferritin (μg/L)	63.0 ± 140.8	78.3 ± 198.5
Transferrin (mg/L)	241.7 ± 60.6	269.3 ± 66.3
Transferrin saturation (%)	9.9 ± 8.85	10.4 ± 6.7
Iron levels (μg/L)	27.6 ± 23.7	39.5 ± 26.3
CPR (mg/L)	52.8 ± 48	18.1 ± 33.5

The array of laboratory testing that may be pertinent in the initial evaluation of anemia in EB patient should include:

1. Complete blood count (CBC):
  Includes hemoglobin, hematocrit, mean corpuscular volume (MCV), mean corpuscular hemoglobin (MCH) and mean corpuscular hemoglobin concentration (MCHC).
2. Iron profile:
  Includes serum iron, ferritin, total iron-binding content (TIBC), transferrin saturation (TSat), and soluble transferrin receptor (sTfR).
3. Reticulocyte count
4. C-Reactive Protein (CRP).

##### Complete blood count

A CBC measures the amounts of red, white blood cells and platelets, along with the hemoglobin and hematocrit values. Red blood cell indices—MCV, MCH and MCHC—which describe the size of red blood cells and their hemoglobin content, are reported along with the red blood cell distribution width (RDW), which measures the amount of variation in the sizes of red blood cells. Along with the hemoglobin and hematocrit, MCV can determine the classification of anemia as microcytic anemia with MCV below the normal range, normocytic anemia with MCV within the normal range, macrocytic anemia with MCV above the normal range [22]. Microcytic anemia is typically associated with iron deficiency, and anemia of chronic disease. The severity of anemia is based on the patient’s hemoglobin/hematocrit level [21]. Reticulocyte count serves as an estimate of bone marrow red blood cell output [21].

##### Iron profile

Total serum iron is a measure of the ferric (Fe3+) ions bound to serum transferrin. There is significant variation of iron levels due to multiple factors, and therefore the serum iron is a poor marker of iron status [22].

Ferritin is an intracellular iron storage protein whose levels are indicative of the body's total iron stores. A reduced ferritin level is the most specific indicator of iron deficiency, as there are no other major causes of hypo-ferritinemia. However, inflammation or liver disease can elevate serum ferritin, masking a concomitant iron deficiency. An elevated serum ferritin is classically a marker of iron overload, though ferritin is also an acute phase reactant and can be non-specifically elevated with alcohol intake, liver disease or chronic inflammation [22]. Serum ferritin is the most used test for diagnosing iron deficiency, with proposed cut-off values ranging from 15 to 100 ng/mL. Based on a systematic review of 55 studies, a ferritin threshold value of < 45 ng/mL has a sensitivity for iron deficiency of 85% (95% confidence interval [CI] 82–87%) with a specificity of 92% (95% CI 91–94%) [23]. The ferritin level may be misleading in the presence of acute or chronic inflammation as ferritin is also an acute phase reactant and thus one cannot exclude iron deficiency as the cause of anemia when the serum ferritin is normal or even elevated in the presence of an inflammatory process [24, 25]. In patients with chronic inflammatory conditions like EB, inflammatory bowel disease, and chronic kidney disease, ferritin levels may not accurately reflect body iron stores. In these situations, other clinical tests, such as the serum iron, transferrin saturation, soluble transferrin receptor (sTfR), sTfR /log ferritin or C-reactive protein, may be useful adjunctive tests to assist in the diagnosis anemia [26].

Transferrin is a transport protein that binds to iron in plasma. This is proportional to the total iron binding capacity (TIBC), the total amount of iron that can be bound to serum transferrin. An elevated transferrin level or TIBC is a marker of iron deficiency; a reduced transferrin / TIBC may occur in the context of an acute phase reaction, chronic disease, or iron overload [22].

The transferrin saturation (TSat) is the percentage of transferrin that is bound to iron. An elevated transferrin saturation can be an indicator of iron overload, such as due to haemochromatosis, multiple transfusions and iron-loading anemias. A reduced transferrin saturation is a marker of iron deficiency, though can also occur with chronic inflammatory disease [22].

The soluble transferrin receptor (sTfR) is rarely performed in clinical practice. Its main utility is in differentiating iron deficiency from anaemia of chronic disease, which can be difficult to distinguish based on standard iron studies. In iron deficiency, the sTfR level will be increased, and it is unaffected by inflammation. Haemolysis or dyserythropoiesis will also cause a raised level. The sTfR level is an indirect measure of erythropoiesis but the limitations are that it is not as reliable as ferritin, and it is not yet widely available in clinical laboratories and its usefulness has been limited by high cost, variability in methods, and different reference ranges [22].

Hepcidin, a liver-derived peptide hormone, is a key regulator of systemic iron homeostasis, and its unbalanced production contributes to the pathogenesis of a spectrum of iron disorders. High hepcidin levels cause iron blockade and anemia in chronic disease. Low hepcidin levels may help distinguish patients with iron deficiency anemia versus anemia of chronic inflammation and patients who may benefit most from iron or erythropoiesis stimulating agents, but more work is needed to understand the clinical utility of hepcidin assays [27].

Interpretation of iron tests are summarized in Table 4 [28].

**Table 4:** Interpretation of Iron studies [28]

	Iron deficiency anemia	Anemia of chronic disease	Iron overload
Hb	Reduced	Reduced	Normal
MCV	Low	Low or low-normal	Normal
Serum iron	Decreased	Decreased	Increased
Transferrin or TIBC	Increased	Decreased	Decreased
Transferrin saturation	Decreased	Normal/decreased	Increased
Soluble transferrin receptor	Increased	Normal	Decreased
Ferritin	Usually < 15	> 200 usually increased	Increased
CRP	Normal	Increased	Normal

### Recommendation 5

#### What are the treatment options to manage anemia in EB patients?

1. Dietary measures should be offered as part of the management of anemia in all EB patients.
2. Optimization of iron-rich food according to geographic location should be offered as part of the management of anemia in all EB patients.
3. Oral iron preparation that is readily available in each geographic area and that is tolerated by the patient should be the iron of choice.
4. Oral iron supplements should be administered every other day to maximize the absorption and minimize the side effects for all patients with mild to moderate anemia.
5. Oral iron should be administered for at least 4 weeks before assessing clinical benefit.

##### Dietary measures

EB patients have increased nutritional requirements because of blistering, chronic wounds, infection, and loss of exudates, and nutritional intake might be compromised because of oropharyngeal blistering and strictures, resulting in malnutrition in many patients [29, 30]. The nutritional impairment in patients with EB is directly related to the severity of the associated problems, i.e., the more severe the EB type, the more extensive the nutritional impairment. Children and adolescents with JEB and RDEB as well as EBS-generalized severe have a significant risk of nutritional deficits [29, 30]. Nutritional compromise occurs early in children with RDEB and therefore may require interventions as of the first year or two of life [20]. The goals of nutritional interventions in children and adolescents with EB are to minimize nutritional deficiencies, improve bowel function, improve feeding time, enhance the immunological status, optimize wound healing, build-up normal body composition and promote proper growth, as well as pubertal and sexual developments. Considering the grave prognosis of some EB types, these goals should be modified according to the patient's situation, with emphasis on the quality of life [7, 29]. Feeding via gastrostomy should be initiated before the onset of malnutrition to improve growth recovery, and before the age of 10 to allow pubertal development, which has a positive psychological impact [31]. Recommendations and examples of iron rich foods to correct anemia in EB patients, as suggested by consensus statements 5-1 and 5-2, are summarized in Table 5 [32]. The recommended dietary allowances (RDAs) for iron are summarized in Table 6 [32].

**Table 5:** Summary of Iron rich food [32]

	Portion size (g)	Iron content (mg)
**Heme iron foods**		
Lamb (lean roasted)	100	2.7
Pork, lean, roasted	100	1.2
Chicken-breast meat	100	0.5
Chicken-leg meat	100	1
Lasagne	400	2.8
Bolognaise sauce	240	3.4
Cottage or Shepherd’s pie		
(meat and vegetable pie)	300	3.6
Steak and kidney pie	200	5
Liver pate	30	2
Corned beef (1 slice)	40	1.2
Meatballs (2 cooked)	190	15.8
Beef burger (1 fried)	40	1.2
Sausages (2 fried)	80	1.3
Sausage roll (1small)	100	1.2
Ham (average slice)	23	0.3
Salmon (fresh cooked)	100	0.8
Salmon (tinned)	100	1.4
Sardines/pilchards (tinned)	100	2.9
Fish paste	25	2.7
Trout	100	1
Tuna (tinned)	50	0.7
Prawns	100	1.1
Mussels (1 = 7 g)	100	7.7
Anchovies	50	2
Mackerel	100	1.2
**Non-heme iron**		
Boiled egg × 1	60	1
Egg yolk, large (1)	–	0.6
Omelet (2 eggs)	120	2
Tofu (steamed and fried)	100	3.5
Lentils (boiled)	100	2.4
Chickpeas (boiled)	100	2.1
Broad beans	100	1.6
Baked beans	100	1.4
Peas	85	1.4
Dark green vegetables (spinach, kale, broccoli, watercress)	75	1
Weetabix whole grain cereal (1 biscuit)	20	2.3
Rice Crispies	30	2.0
Cornflakes	30	2.0
Whole wheat bread (1 slice)	30	0.8
White bread (1 slice)	30	0.5
Dried apricots (× 4)	32	1.0
Raisin/sultanas (1tbs)	30	1.1
Almonds, hazelnuts, peanuts, walnuts	30	1.0
Pine nuts	30	1.7
Peanut butter (spread on toast)	20	0.4
Chocolate (standard bar) dark	50	1.2
Chocolate (standard bar) milk	50	0.8
Cocoa powder (1 tsp)	6	0.6

Iron from food comes in two forms: heme and non-heme. Heme is found only in animal flesh like meat, poultry, and seafood. Non-heme iron is found in plant foods like whole grains, nuts, seeds, legumes, and leafy greens. Heme iron food have better bioavailability than non-heme iron sources

**Table 6:** Recommended dietary allowances (RDAs) for iron [32]

Age	Male (mg)	Female (mg)	Pregnancy (mg)	Lactation (mg)
Birth to 6 months	0.27*	0.27*		
7–11 months	11	11		
1–3 years	7	7		
4–8 years	10	10		
9–13 years	8	8		
14–18 years	11	15	27	10
19–50 years	8	18	27	9
> 50 years	8	8		

*Adequate intake

##### Enteral iron supplementation

Enteral iron supplements are widely used in patients with EB. Although available in a variety of different preparations, they are commonly associated with gastrointestinal upset (either constipation or, less commonly, diarrhea) and are often unpalatable. As a result, adherence to treatment is often limited. The effectiveness of enteral iron supplementation may also be limited by reduced absorption from the gut, either as a result of hepcidin-induced decreased enteral iron absorption, iron deficiency itself or possibly from a direct effect of EB on the gut [4]. In a study enteral iron absorption challenges in 12 patients with RDEB from the USA over a 5-year period, patients were given a dose of 2 mg elemental iron/kg by mouth or via G-tube in the form of ferrous sulfate liquid after a 6- to 8-h fast. Baseline hemoglobin, ferritin, soluble transferrin receptor, and inflammatory markers were collected within days to 3 months of the iron challenge, and serum iron was collected after intervals of 2–4 h. Inflammatory markers were tested within 3 months of the iron absorption tests, while baseline hemoglobin and iron studies were most often done within 3 days preceding the iron absorption test. 9/12 patients did not show increased iron absorption, and all patients had elevated ESR and CRP, low serum albumin and hemoglobin levels [9]. The three patients with challenges with elevated iron absorption also had elevated soluble transferrin receptor (STFR)/log ferritin, as well as elevated ESR and CRP, but these inflammatory markers were less elevated than those in non-absorbers, consistent with inflammation causing a decrease in iron absorption [25]. Poor gastrointestinal iron absorption may be an important factor in failure to improve anemia in RDEB enterally [9].

According to the WHO, for the general population the recommended dosage of elemental iron required to treat iron deficiency anemia in adults is 120 mg per day for at least three months; the dosage for children is 3–6 mg per kg per day, up to 60 mg per day [21]. First-line treatment is oral therapy with ferrous iron salts, but a substantial proportion of patients suffer from gastrointestinal side-effects, resulting in non-adherence and treatment failure [33]. Gastrointestinal symptoms most likely result from a combination of two factors: (i) free radical generation through iron-induced redox cycling in the gut lumen and at the mucosal surface which can promote inflammation and (ii) changes to the microbiota composition or metabolism [33]. Ferrous sulfate remains the most prescribed oral iron therapeutic, but several formulations exist and have mainly been investigated for the treatment of iron deficiency [33, 34]. Dosage recommendations are based on each preparation’s content of elemental iron in milligrams. Frequently used forms of iron in supplements include ferrous and ferric iron salts, such as ferrous sulfate, ferrous gluconate, ferric citrate, and ferric sulfate. Because of its higher solubility, ferrous iron in dietary supplements is more bioavailable than ferric iron [35]. High doses of supplemental iron (45 mg/day or more) may cause gastrointestinal side effects, such as nausea and constipation. Other forms of supplemental iron, such as heme iron polypeptides, carbonyl iron, iron amino-acid chelates, polysaccharide-iron complexes and sucrosomial iron (a new oral iron preparation containing ferric pyrophosphate protected by phosoholipid bilayer plus a sucrester matrix) might have fewer gastrointestinal side effects, better tolerability and higher biodisponibility than ferrous or ferric salts [35, 36].

The different forms of iron in supplements contain varying amounts of elemental iron. For example, ferrous fumarate is 33% elemental iron by weight, whereas ferrous sulfate is 20% and ferrous gluconate is 12% elemental iron [35]. Elemental iron is listed in the Supplement Facts panel, so consumers do not need to calculate the amount of iron supplied by various forms of iron supplements [35].

There is no strong evidence that any of the available oral formulations are more effective or better tolerated than the others; patient tolerance should be the guide [35–38]. Oral iron formulations and dosing are summarised in Table 7. Some studies suggest that lower dosing or an every-other-day single morning dose may improve tolerability and absorption [39, 40]. For patients with mild to moderate anemia, oral iron supplements should be administered every other day in a single morning dose to maximize absorption and minimize side effects [40].

**Table 7:** Oral iron formulations and dosing [35, 36]

Formulation	Dosage forms (elemental iron)
Ferrous fumarate	Tab 90 (29.5) mg, 325 (106) mg, 456 (150) mg
Ferrous gluconate	Tab 240 (27) mg, 256 (28 mg), 325 (36) mg
Ferrous sulfate	Drops and oral solution;75 (15) mg per mL Elixir and liquid: 220 (44) mg per mL Syrup: 300 (60) mg per mL Tablet 300 (60) mg, 325 (65) mg Extender-release tablets: 140 (45) mg, 160 (50) mg, 325 (65) mg
Ferric sodium EDTA/ferric ammonium citrate	Spray: 5 mg of iron per 4 sprays
Polysaccharide-iron complex and ferrous bisglycinate chelate	Capsule elemental iron (50, 150 mg with or without 50 mg vitamin C) Elixir: elemental iron (100 mg per 5 mL)

Taking iron supplements with food or using enteric-coated formulations may improve tolerability but decrease absorption. Vitamin C co-administration is commonly recommended to improve oral absorption, although the evidence supporting this practice is limited [26, 41]. Special considerations about the proper administration of oral iron, trying to minimize adverse effects and improve its absorption, are summarized in Table 8 [33].

**Table 8:** General recommendations to optimize oral iron treatment absorption [33, 35, 40, 41]

Iron should not be given with food or with empty stomach, should be given half hour post meal	
Iron should be taken separately from calcium-containing foods and beverages (milk), calcium supplements, cereals, dietary fiber, tea, coffee, and egg	
Iron should be given 2 h before or 4 h after ingestion of antacids	
Coadministration of 250 mg of ascorbic acid or half glass of orange juice with iron to enhance its absorption	
Providing iron supplements on alternate days and in single doses optimises iron absorption and compliance may also be higher with this posology	

An increase in hemoglobin of 1 g per dL after one month of treatment shows an adequate response to treatment in non-EB patients [42], but this increase may be much less in EB patients although there are no data to support this. Anemia should correct in about 3–6 months. It is recommended to continue oral iron for 3–6 months after anemia corrects to replenish iron stores [21, 42].

##### Intravenous iron

Intravenous iron should be considered when there is inadequate response to enteral iron, intolerance to enteral iron therapy, or when a patient fails an enteral iron absorption challenge [4, 9, 43]. Newer and safer preparations of intravenous iron, including iron (III) hydroxide-sucrose complex [13] and ferric gluconate complex [8, 44], are preferable to iron dextran [13, 44], which is associated with a higher risk of anaphylaxis. Giving intravenous iron has been shown to improve hemoglobin levels in patients with EB, to increase general well-being, and to reduce reliance on blood transfusion, although patient numbers in these studies have been small [8, 13, 43]. Intravenous access to administer iron therapy, or pain associated with intramuscular iron dextran injections, may limit the usefulness of parenteral iron for some patients with EB. However, a number of these patients would be otherwise dependent on blood transfusion for their anemia and would also have all the associated problems of gaining intravenous access in the context of extensive skin involvement and scarring [4, 9, 14]. A recent study of 43 adults with anemia and severe EB demonstrated that periodic IV ferric carboxymaltose (FCM) infusions were safe and effective in increasing Hb levels, and easy to administer as a full dose of required iron was able to be delivered in 15 min infusion. The authors recommended IV FCM as first-line treatment for anemia with Hb levels below 100–110 g/L (10–11 g/dL) in this patient group, repeated 3–4 monthly according to laboratory and clinical response [45]. Caution must be applied regarding multiple/frequent ferric carboximaltose dosing, due to its relation to chronic hypophosphatemia and osteomalacia [46]. As patients with severe forms of EB are likely to need multiple doses per year, keeping this side effect in mind and applying monitoring strategies (parathyroid hormone, bone profile labs) at baseline and post infusion might be an aspect to consider.

Available IV -IM iron supplementation options and dosing are summarized in Table 9. Due to difficulty in IV access, adhering to an “ideal schedule” may be challenging. Taking advantage of IV access (i.e., when there is an IV for other procedures) and giving the largest possible dose at that time may be reasonable.

**Table 9:** Intravenous iron formulations and dosing

Intravenous iron preparation	Maximum recommended dose	Duration of infusion
Low molecular weight iron dextran*	Test dose: administer prior to start iron dextran therapy and observe for 15–30 min Infants ≥ 4 months and < 10 kg: 10 mg (0.2 ml) Children 10–20 kg: 15 mg (0.3 ml) Children > 20 kg and adults 25 mg (0.5 ml)	–
	Therapeutic dose IV 20 mg/kg, max 1000 mg/dose	1 h
	Therapeutic dose IM Infants ≥ 4 months < 5 kg:25 mg (0.5 ml)/day Children 5–10 kg: 50 mg (1 mL)/day Children > 10 kg and adults 100 mg (2 mL)/day	–
Iron sucrose	Test dose: not necessary Children ≥ 2 years of age: 5–7 mg/kg/dose; maximum initial dose: 100 mg/dose. Maintenance dosing 5 to 7 mg/kg/dose every 1 to 7 days until total replacement dose achieved; maximum single dose: 300 mg/dose Adults: 100–300 mg per dose (frequency and duration of therapy may vary); repeated until hematologic parameters or total iron requirements are met	30–90 min
	Adults: 500 mg once weekly for 2 weeks	4 h
Ferric carboxymaltose	Safety and efficacy in children not established	
	Adults: < 50 kg 15 mg/kg IV in 2 doses separated by at least 7 days Not to exceed cumulative dose 1500 mg per course Adults: ≥ 50 kg 750 mg IV in 2 doses separated by at least 7 days; not to exceed cumulative dose of 1500 mg per course Alternatively, may administer 15 mg/kg IV as a single dose; not to exceed 1000 mg	Minimum 15 min (100 mg/min)
Ferumoxytol	Safety and efficacy in children not established	
	Adults: 510 mg × 2 doses 3 to 8 days apart 1.02 g over ~ 30 min as a single dose	15–30 min
Sodium ferric gluconate	Children ≥ 6 years of age: 1.5 mg/kg elemental Fe IV do not exceed 125 mg	1 h
	Adults: 125 mg q treatment × 8 doses	

Commonly available concentrations are listed, but other concentrations may be available, and some brands may have been reformulated. Always refer to the latest available information on specific products. Dosing from UpToDate and Lexicomp

*Can also be given intramuscularly

The Ganzoni Equation for Iron Deficiency Anemia can be used to calculate iron deficit for dosing IV iron as below:

$${\text{Total}}\,{\text{Iron}}\,{\text{Deficit }} = {\text{Weight}}\,{\text{in}}\,({\text{kg }}) \times \, [{{\text{Target}}\,{\text{ Hb }}{-}{\text{ Actual}}\,{\text{Hb}}\,{\text{in}}\,({\text{g}}/{\text{dL}}}) ] \, \times { 2}.{4 } + \, *{\text{Iron}}\,{\text{stores}}\,{\text{in}}\,({\text{mg}})$$

*For iron stores use 500 if Wt > 35 kg; 15 mg/kg if Wt < 35 kg.

The number of doses required will depend on the total iron deficiency calculated with the Ganzoni equation.

##### Erythropoietin

The potential for erythropoietin to improve EB-associated anemia has also been explored in a few small studies where it (or an analogue, darbepoetin alfa) was administered along with intravenous iron [2, 8, 43]. Although low endogenous erythropoietin levels have been found in some anemic EB patients, elevated levels have been found in others [2, 43]. Despite anemia driving the increased secretion of erythropoietin, animal studies have shown reduced erythropoietin levels associated with protein depletion [47] suggesting that this can also be seen in patients with EB. Cytokines found in chronic inflammation may also decrease the kidney’s production of erythropoietin. Many anemic EB patients also have significant nutritional compromise and low serum albumin levels [29], and this may contribute to the low observed erythropoietin levels in some patients [2, 43]. However, even in patients with elevated endogenous erythropoietin levels, a combination of intravenous ferric gluconate or intramuscular iron dextran, given with weekly subcutaneous darbepoetin alfa, improved energy levels and hemoglobin measurements in four patients with RDEB [8]. Based on the small number of patients reported, the additional benefit of erythropoietin over iron alone is difficult to ascertain. Drawbacks to treatment with erythropoietin include the need for intravenous or subcutaneous administration and high cost. Darbepoetin alfa has the advantage of a longer half-life, meaning that it is usually given weekly rather than two or three times per week, although its cost is higher. There is insufficient evidence to support the use of erythropoietin in the absence of erythropoietin deficiency and/or renal failure [2, 43]. Further studies, with larger patient numbers and comparison of parenteral iron alone versus parenteral iron and erythropoietin, are clearly needed [9].

##### Blood transfusion

Even with attempts to correct iron deficiency as described above, many patients with severe forms of EB still require blood transfusion [4, 9, 14]. This is often considered when the Hb level reaches around 7–8 g/dL or at higher levels if the patient is significantly symptomatic, although there exist no clear, evidence-based protocols to support this approach. Concerns with blood transfusion include difficulties obtaining intravenous access in severe EB, transfusion reactions, and the risks of blood-borne infection. As problems of iron overload have been seen in patients with repeated transfusions, it is possible that EB patients who receive repetitive transfusions may potentially have exacerbations of EB-associated cardiomyopathy, but this has not been fully elucidated [4, 48].

### Recommendation 6

#### What should be the outcome of treating anemia in EB patients?

1. Improvement of symptoms.
2. Improvement of laboratory parameters.

EB patients sometimes find it difficult to comply with recommended oral iron supplementation. They also have decreased iron absorption, increase iron loss, and inflammation, which may contribute to lower-than-expected improvements in hemoglobin in comparison to non -EB patients. Therefore, close monitoring every 3 months of clinical symptoms and every 6 months of laboratory parameters, especially in RDEB and JEB generalized severe, is recommended, as blood samples are difficult to obtain in this population [9, 14, 19].

## Conclusions

Our consensus guidelines suggest that anemia is most common in patients with RDEB and JEB, but poorly studied in patients with EB in general. In addition to asking patients about symptoms and monitoring overall status, screening labs are needed in the high-risk populations. Laboratory testing that may be pertinent during the initial evaluation of anemia in EB patient should include CBC, reticulocyte count, ferritin, CRP levels and TIBC; in patients with severe generalized forms of EB, screening should be done at diagnosis and repeated every 6 months. For mild to moderate forms of EB, screening blood work should be done at 1 year of age and then repeated yearly or when symptomatic. Treatment can start with oral iron supplementation but may need to be escalated to IV iron or blood transfusion based on response and symptoms. IV iron should be administered if Hb levels are > 80–100 mg/dl and, in general, blood transfusion should be administered if Hb is < 80 mg/dl. The role of erythropoietin is unclear or not recommended unless there is documented renal failure and/or erythropoietin deficiency.

In conclusion, anemia in EB is multifactorial and challenging to diagnose and treat. This consensus guideline offers a practical approach about when and how to diagnose, monitor, and treat anemia in EB patients.

There are currently few data that offer providers evidence-based guidance, and future research is needed.

### Further research

Prospective studies on epidemiological, clinical characteristics and management are required to better understand and treat anemia in EB.

### Updating procedure and dissemination

The consensus guidelines will be updated every 3–5 years or earlier if there is a significant breakthrough in EB anemia treatment from the publication date. We recommend a literature search to see whether a full review is warranted at any stage.

DI aims to ensure that the EB CPG address the needs of patients internationally. The consensus guidelines will be presented at the international DEBRA Congresses. DI recommends that implementation of these recommendations should be monitored and evaluated through audits. The completion of a current practice audit, followed by the CPG pre-implementation survey (https://surveyhero.com/c/aabc0100) and post-implementation survey are highly recommended for best practice.

## Supplementary Information

**Additional file 1.** Appendix 1. Anemia in epidermolysis bullosa: Patient/Family member survey.

**Additional file 2.** Appendix 2. Anemia in epidermolysis bullosa: Healthcare provider survey.

## Data Availability

Not applicable.

## Abbreviations

EB: Epidermolysis bullosa
EBS: Epidermolysis bullosa simplex
JEB: Junctional epidermolysis bullosa
RDEB: Recessive dystrophic epidermolysis bullosa
CPG: Clinical practice guidelines
HCP: Health care providers
Hb: Hemoglobin
CBC: Complete blood count
CRP: C-reactive protein
ESR: Erythrocyte sedimentation rate
TIBC: Total iron binging capacity
sTfR: Soluble transferrin receptor
FCM: Ferric carboxymaltose
