# Supplementary material for: Consensus guidelines for diagnosis and management of anemia in epidermolysis bullosa
Source: Orphanet J Rare Dis. 2023 Feb 23;18:38. doi: 10.1186/s13023-022-02448-w (PMC9948325; doi:10.1186/s13023-022-02448-w)
Supplement: Supplementary file 1 — Additional file 1. Appendix 1. Anemia in epidermolysis bullosa: Patient/Family member survey. [file 13023_2022_2448_MOESM1_ESM.docx]

**APPENDIX 1: ANEMIA IN EPIDERMOLYSIS BULLOSA (EB): PATIENT/FAMILY MEMBER SURVEY**

| **ANEMIA IN EPIDERMOLYSYS BULLOSA: Patient & Family Member Survey**  Q1: Please select your role:   1. Patient with EB 2. Family member of patient with EB   Q2: Do you worry about having anemia? If yes, please explain why you worry about  having anemia.   1. Yes 2. No 3. Not sure   Q3: Do you know when your blood count drops (when you become anemic)?   1. Yes 2. No 3. Not sure   Q4: How do you know when you become anemic?   1. Wounds heal slower or do not heal 2. I feel more tired 3. My heart races 4. I get short of breath 5. I develop a low mood   Q5: Have you ever received any treatment for anemia?   1. Yes (please proceed to question 6) 2. No (please proceed to question 11) 3. Not sure (please proceed to question 11)   Q6: How was your anemia treated? Please mark all that apply   1. Diet 2. Oral iron 3. Intravenous iron 4. Intramuscular iron 5. Transfusion   Q7: Did you experience any problems with the treatment you received?   1. Yes 2. No 3. Not sure   Q8: What problems did you experience with the treatment you received?   1. Stomach upset 2. Stomach pain 3. Nausea or vomiting 4. Constipation 5. Diarrhea 6. Chest or throat pain 7. Allergic reaction   Q9: When you have anemia, do you have a preferred treatment?   1. Oral iron 2. Intravenous iron 3. Intramuscular iron 4. Other: Please specify   Q10: Do you have a preferred oral iron medication?   1. Yes. If yes, please specify 2. No   Q11: The most challenging part of managing my anemia is…(Free Text)  Q12: I think anemia guidelines are important because…(Free Text)  Q13: Please specify the type of EB you or your relative has (Free Text) |
| --- |
