# Supplementary material for: Consensus guidelines for diagnosis and management of anemia in epidermolysis bullosa
Source: Orphanet J Rare Dis. 2023 Feb 23;18:38. doi: 10.1186/s13023-022-02448-w (PMC9948325; doi:10.1186/s13023-022-02448-w)
Supplement: Supplementary file 2 — Additional file 2. Appendix 2. Anemia in epidermolysis bullosa: Healthcare provider survey. [file 13023_2022_2448_MOESM2_ESM.docx]

**APPENDIX 2: ANEMIA IN EPIDERMOLYSIS BULLOSA (EB): HEALTHCARE PROVIDER SURVEY**

| **ANEMIA IN EPIDERMOLYSYS BULLOSA: Healthcare Professional Survey**  Q1: Please select your role:   1. Pediatrician 2. Dermatologist 3. Hematologist 4. Nutritionist 5. Internist 6. Family Physician 7. Other (Please Specify)   Q2: Which country do you work in? (Free Text)  Q3: How many years of experience do you have working with EB patients?   1. 1-10 years 2. 10-20 years 3. More than 20 years   Q4: How many EB patients do you see per month?   1. 1-10 years 2. 10-20 years 3. More than 20   Q5: How often do you do clinical and laboratory screening for anemia in EB patients? Please select  the type of EB (EBS, JEB, DDEB, RDEB) and frequency of screening.   1. Monthly 2. Every 3 months 3. Every 6 months 4. Annually 5. Only when symptomatic 6. Other   Q6: At what age do you start laboratory screening for anemia in EB? Please select the type of EB (EBS, JEB, DDEB, RDEB) and patient age.   1. 1 year of age 2. 2-5 years of age 3. 6-10 years of age 4. >10 years of age 5. Other   Q7: When you request bloodwork in EB patients to diagnose anemia, which tests are ordered? Please select all that apply.   1. Complete blood count 2. Iron 3. Serum ferritin 4. Transferring saturation 5. Total Iron Binding Capacity (TIBC) 6. ESR 7. Methylmalonic acid 8. Homocysteine 9. Ceruloplasmin 10. Pyridoxine 11. Others (Please specify)   Q8: In an EB patient with anemia, which laboratory parameter would you consider to be the most important to start treatment?   1. Hemoglobin 2. iron 3. Hemoglobin and iron 4. Serum transferrin 5. Ferritin and hemoglobin 6. Transferrin and hemoglobin   Q9: In an EB patient with anemia, which laboratory parameter do you consider to be the most reliable to monitor response to treatment?   1. Hemoglobin 2. Iron 3. Transferrin saturation 4. Ferritin 5. Reticulocytes 6. Total Iron Binding Capacity 7. Hematocrit   Q10: What is your target hemoglobin for EB patients?   1. 70g/L 2. 80g/L 3. 90g/L 4. 100g/L 5. 110g/L 6. 120g/L 7. Other   Q11: How do you define mild anemia in EB patients?   1. 100-120g/L 2. 80-100g/L 3. <80g/L 4. Other (Please specify)   Q12: How do you treat mild anemia?   1. Dietary measures 2. Oral iron 3. Iron transfusion 4. RBC Transfusion 5. Other (Please specify)   Q13: How do you define moderate anemia in EB patients?   1. 100-120g/L 2. 80-100g/L 3. <80g/L 4. Other (Please specify)   Q14: How do you treat moderate anemia?   1. Dietary measures 2. Oral iron 3. Iron transfusion 4. RBC Transfusion 5. Other (Please specify)   Q15: How do you define severe anemia in EB patients?   1. <90g/L 2. <80g/L 3. 70-80g/L 4. <70g/L 5. <60g/L 6. <40g/L 7. Other (Please specify)   Q16: How do you treat severe anemia?   1. Dietary measures 2. Oral iron 3. Iron transfusion 4. RBC Transfusion 5. Other (Please specify)   Q17: When you decide to start treatment for anemia, you rely most on:   1. Clinical symptoms 2. Laboratory parameters 3. Both 4. Other (Please specify)   Q18: What are the treatment options for anemia that you recommend most frequently?   1. Dietary measures 2. Oral iron 3. Iron transfusion 4. RBC Transfusion 5. Other (Please specify)   Q19: What type of oral iron do you prefer to use for EB patients?   1. Perric pyrophosphate 2. Ferrous gluconate 3. Ferrous sulfate 4. Ferrous sulfate dried 5. Ferrous fumarate 6. Ferrous carbonate anhydrous 7. Polysaccharide iron complex 8. Carbonyl iron 9. Other (Please specify)   Q20: What are the benefits to treatment with oral iron?   1. Experience with better results 2. Best tolerance and absorption 3. Tolerability 4. Most commonly used in country 5. Cost and accessibility 6. Compliance 7. Hospital protocol   Q21: What type of IV iron do you prefer to use for EB patients?   1. Ferric carboxymaltose 2. Ferric gluconate 3. Iron sucrose 4. Iron isomaltoside 5. Iron dextran   Q22: How often do you administer IV iron in EB patients?   1. Every 3 months 2. Every 6 months 3. Yearly 4. When symptomatic 5. Whenever IV access is available (Ex: Other procedures) 6. Other (Please specify)   Q23: What do you think plays the most important role in the development of anemia in EB patients?   1. Iron deficiency due to poor iron absorption 2. Low iron intake 3. Inflammation 4. Blood loss through wounds 5. Other (Please specify)   Q24: What should be the primary outcome of treatment anemia in EB patients?   1. Corrected levels of hemoglobin according to patient’s age 2. Improved iron parameters 3. Improvement of clinical symptoms from baseline 4. Fatigue scores 5. Other (Please specify)   Q25: In your country, are there any unique treatments or recommendations that you give your EB patients with anemia? (Free text)  Q26: Are there any special resources that you use for managing anemia in EB patients?   1. No special resources 2. EB dietician 3. Hematologist counselling 4. Other (Please specify) |
| --- |
